# Supplementary material for: Perspectives on innovation of the diagnostic process in general practice: Q-methodological study
Source: BMC Prim Care. 2026 Feb 9;27:163. doi: 10.1186/s12875-026-03200-0 (PMC13112758; doi:10.1186/s12875-026-03200-0)
Supplement: Supplementary file 1 — Supplementary Material 1. [file 12875_2026_3200_MOESM1_ESM.docx]

**Supplements**

| **Supplementary Table 1** Search terminology | |
| --- | --- |
| **Search criteria** | **Terminology** |
| Perspectives | ‘perspective’, ‘perspectives’, ‘opinion’, ‘opinions’, ‘attitudes’, ‘attitude’, ‘viewpoint’, ‘viewpoints’, ‘views’, ‘view’, ‘point of view’, ‘perception’, ‘belief’, ‘approach’ |
| Multidisciplinary stakeholders | ‘doctor’, ‘doctors’, ‘physician’, ‘physicians’, ‘general practitioner’, ‘general practitioners’, ‘family doctor’, ‘GP’, ‘G.P.’, ‘family practitioner’, ‘family physician’, ‘nurse’, ‘family nurse’, ‘nurse practitioners’, ‘radiologist’, ‘radiologists’, ‘clinical chemist’, ‘clinical chemists’, ‘patient’, ‘patients’ |
| Diagnostics | ‘diagnosis’, ‘diagnostics’, ‘examination’, ‘diagnosing’, ‘diagnostic imaging’, ‘point of care’, ‘point-of-care’, ‘point of care testing’, ‘diagnostic test’, ‘diagnostic tests’, ‘diagnostic process’, ‘diagnostic tool’, ‘diagnostic tools’ |
| Innovation | ‘innovation, ‘innovative’, ‘new’, ‘improving’, ‘improved’, ‘improvements, ‘improvement’, ‘invention’, ‘inventions’, ‘inventive’, ‘innovational’ |
| General practice domain | ‘General practice’, ‘primary care’, ‘primary healthcare’, ‘primary health care’, ‘outpatient care’, ‘family care’, ‘family health care’, ‘first-line care’, ‘first line care’, ‘first-line health care’, ‘first-line healthcare’, ‘first line health care’, ‘first line healthcare’ |
| Qualitative | ‘qualtitative’, ‘narrative’, ‘descriptive’ |

**Supplementary Box 1** Search strings for Pubmed and Embase

#### Pubmed

(((((((((((((((perspective[Title/Abstract]) OR (perspectives[Title/Abstract])) OR (opinion[Title/Abstract])) OR (opinions[Title/Abstract])) OR (attitudes[Title/Abstract])) OR (attitude[Title/Abstract])) OR (viewpoints[Title/Abstract])) OR (viewpoint[Title/Abstract])) OR (point of view[Title/Abstract])) OR (perception[Title/Abstract])) OR (belief[Title/Abstract])) OR (approach[Title/Abstract])) AND ((((((((((((((((((((doctor[Title/Abstract]) OR (doctors[Title/Abstract])) OR (physician[Title/Abstract])) OR (physicians[Title/Abstract])) OR (general practitioner[Title/Abstract])) OR (general practitioners[Title/Abstract])) OR (family doctor[Title/Abstract])) OR (gp[Title/Abstract])) OR (g.p.[Title/Abstract])) OR (family practitioner[Title/Abstract])) OR (family physician[Title/Abstract])) OR (nurse[Title/Abstract])) OR (family nurse[Title/Abstract])) OR (nurse practitioners[Title/Abstract])) OR (radiologist[Title/Abstract])) OR (radiologists[Title/Abstract])) OR (clinical chemist[Title/Abstract])) OR (clinical chemists[Title/Abstract])) OR (patient[Title/Abstract])) OR (patients[Title/Abstract]))) AND (((((((((((((diagnosis[Title/Abstract]) OR (diagnostics[Title/Abstract])) OR (examination[Title/Abstract])) OR (diagnosing[Title/Abstract])) OR (diagnostic imaging[Title/Abstract])) OR (point of care[Title/Abstract])) OR (point-of-care[Title/Abstract])) OR (point-of-care-testing[Title/Abstract])) OR (diagnostic test[Title/Abstract])) OR (diagnostic tests[Title/Abstract])) OR (diagnostic process[Title/Abstract])) OR (diagnostic tool[Title/Abstract])) OR (diagnostic tools[Title/Abstract]))) AND ((((((((((((((general practice[Title/Abstract]) OR (primary care[Title/Abstract])) OR (healthcare[Title/Abstract])) OR (primary healthcare[Title/Abstract])) OR (primary health care[Title/Abstract])) OR (outpatient care[Title/Abstract])) OR (family care[Title/Abstract])) OR (family health care[Title/Abstract])) OR (first-line care[Title/Abstract])) OR (first line care[Title/Abstract])) OR (first-line health care[Title/Abstract])) OR (first-line healthcare[Title/Abstract])) OR (first line health care[Title/Abstract])) OR (first line healthcare[Title/Abstract]))) AND (((qualitative[Title/Abstract]) OR (narrative[Title/Abstract])) OR (descriptive[Title/Abstract]))

Filters applied: full text, 2019-2023, humans, Language: Dutch/English.

975 results

#### Embase

('perspectives':ti,ab,kw OR 'perspective':ti,ab,kw OR 'opinion':ti,ab,kw OR 'opinions':ti,ab,kw OR 'attitude':ti,ab,kw OR 'attitudes':ti,ab,kw OR 'viewpoints':ti,ab,kw OR 'viewpoint':ti,ab,kw OR 'views':ti,ab,kw OR 'view':ti,ab,kw OR 'point of view':ti,ab,kw OR 'perception':ti,ab,kw OR 'belief':ti,ab,kw OR 'approach':ti,ab,kw)

AND

('doctor':ti,ab,kw OR 'doctors':ti,ab,kw OR 'physician':ti,ab,kw OR 'physicians':ti,ab,kw OR 'general practitioner':ti,ab,kw OR 'general practitioners':ti,ab,kw OR 'family doctor':ti,ab,kw OR 'gp':ti,ab,kw OR 'g.p.':ti,ab,kw OR 'family practitioner':ti,ab,kw OR 'family physician':ti,ab,kw OR 'nurse':ti,ab,kw OR 'family nurse':ti,ab,kw OR 'nurse practitioner':ti,ab,kw OR 'radiologist':ti,ab,kw OR 'clinical chemist':ti,ab,kw OR 'clinical chemists':ti,ab,kw OR 'patient':ti,ab,kw OR 'patients':ti,ab,kw)

AND

('diagnosis':ti,ab,kw OR 'diagnostics':ti,ab,kw OR 'examination':ti,ab,kw OR 'diagnosing':ti,ab,kw OR 'diagnostic process':ti,ab,kw OR 'diagnostic imaging':ti,ab,kw OR 'point of care':ti,ab,kw OR 'point-of-care':ti,ab,kw OR 'point-of-care testing':ti,ab,kw OR 'diagnostic test':ti,ab,kw OR 'diagnostic tests':ti,ab,kw OR 'diagnostic tool':ti,ab,kw OR 'diagnostic tools':ti,ab,kw)

AND

('innovation':ti,ab,kw OR 'innovative':ti,ab,kw OR 'new':ti,ab,kw OR 'improving':ti,ab,kw OR 'improvement':ti,ab,kw OR 'improved':ti,ab,kw OR 'improvements':ti,ab,kw OR 'invention':ti,ab,kw OR 'inventions':ti,ab,kw OR 'inventive':ti,ab,kw OR 'innovational':ti,ab,kw)

AND

('general practice':ti,ab,kw OR 'primary care':ti,ab,kw OR 'primary healthcare':ti,ab,kw OR 'primary health care':ti,ab,kw OR 'outpatient care':ti,ab,kw OR 'family care':ti,ab,kw OR 'family health care':ti,ab,kw OR 'first-line care':ti,ab,kw OR 'first line care':ti,ab,kw OR 'first-line health care':ti,ab,kw OR 'first line health care':ti,ab,kw OR 'first line healthcare':ti,ab,kw)

AND

('qualitative':ti,ab,kw OR 'narrative':ti,ab,kw OR 'descriptive':ti,ab,kw)

AND ([dutch]/lim OR [english]/lim) AND [humans]/lim AND [2013-2023]/py

Results: 576

**Supplementary Box 2** Search strings for specific journals

The specific journals were: The New England Journal of Medicine (NEJM) Catalyst, Healthcare: The Journal of Delivery Science and Innovation, and Journal of Medical Internet Research (JMIR). The first journal was searched manually, as it is not indexed in PubMed or Embase. Although the other two journals are included in the databases, our search was broad in scope. Therefore, we conducted an additional search within these journals to ensure relevant articles were not missed. These journals were searched to add other viewpoints to the search, including organizational, social, ethical, financial, and political viewpoints.

#### New England Journal of Medicine Catalyst Innovations in Care Delivery

title:'primary AND title:care' OR title:'general AND title:practice'

No filters applied, 35 results.

#### Healthcare: The Journal of Delivery Science and Innovation

Title: 'primary care' OR 'general practice' OR 'diagnostics' OR 'diagnose'

Results filtered for years 2018-2023, 36 results.

#### Journal of Medical Internet Research

Primary care [title/abstract/keyword] AND innovation [title/abstract/keyword] AND ('perspectives' OR 'qualitative' OR 'interview' OR 'perceptions' [title/abstract/keyword]) AND ('diagnosing' OR 'diagnostics' OR 'diagnose' OR 'diagnosis' [title/abstract/keyword])

**Supplementary Box 3** Search strings for media

#### Nexis Uni

Search terms: Innovatie AND huisartsenpraktijk OR eerste lijn
Filters: last 5 years, content type: news. Group duplicates on.

#### Huisarts en Wetenschap

Search terms: innovatie diagnostiek
Filters: years 2019 – 2023

#### Google news

Search terms: innovatie AND huisarts

| **Supplementary Table 2** All 57 Q-sample statements and idealized grid for each perspective of stakeholders on innovation of the diagnostic process in general practice care. |
| --- |

| **#** | **Statement** | **Perspective 1** | **Perspective 2** | **Perspective 3** | **Perspective 4** | **Perspective 5** |
| --- | --- | --- | --- | --- | --- | --- |
|  |  |  |  |  |  |  |
|  |  | Innovation through diagnostic transformation | Innovation in communication | Innovation from a doctor-centered perspective | System reform before innovation: fixing the foundation first | Ambivalence towards innovation |
| **1** | Digital remote diagnostics is convenient for both patient and caregiver. | 2 | 0 | -1 | 2 | 0 |
| **2** | Additional diagnostics should be used primarily based on the patient's request for help. | -1 | 3 | -2 | -1 | -3 |
| **3** | General practitioners need more support in making decisions about relevant diagnostics. | 0 | -2 | 4 | 1 | -1 |
| **4** | The more diagnostic tests are feasible in general practice itself, the better. | -2 | -4 | -1 | -3 | -4 |
| **5** | Doctors can make better diagnostic decisions using Artificial Intelligence. | 1 | 0 | 1 | 2 | 3 |
| **6** | Better communication between general practitioners and medical specialists is necessary to improve the diagnostic process. | 1 | 3 | 0 | 3 | -1 |
| **7** | Patient records must be immediately accessible to primary and secondary care providers. | 2 | 4 | 2 | 4 | 3 |
| **8** | A single national electronic patient record must be developed. | 3 | 4 | 1 | 4 | 2 |
| **9** | There must be more and larger health centers with diagnostic facilities in which general practitioners and specialists work together. | -1 | -1 | -3 | 3 | -2 |
| **10** | The use of Artificial Intelligence systems is a solution to capacity problems in healthcare. | 3 | 0 | -4 | -2 | 3 |
| **11** | There should be more focus on implementing diagnostic innovations in practice rather than on initial development. | -2 | 2 | 0 | 0 | 1 |
| **12** | Clearer agreements must be made on the use of reliable home measurement devices. | 2 | 0 | 1 | 2 | 2 |
| **13** | Regional agreements should be made between general practitioners, hospitals and laboratories on diagnosis requests to avoid double diagnosis. | 1 | 1 | -1 | 0 | 2 |
| **14** | The use of Artificial Intelligence in diagnostic decisions limits the patient's freedom of choice. | -3 | -3 | -4 | -3 | -4 |
| **15** | The patient should have a greater role in deciding whether or not to use diagnostics. | -3 | 1 | -3 | -1 | -2 |
| **16*** | Primary care physicians are reluctant to embrace innovation in diagnostics. | -2 | -2 | -1 | -2 | 0 |
| **17** | Clinical decision rules are useful in determining whether an indication exists for requesting additional diagnostics. | 3 | 3 | 4 | 3 | -1 |
| **18** | The use of ultrasound in general practice improves the diagnostic process. | -1 | -1 | 0 | -2 | -2 |
| **19** | Diagnostic tests in general practice are preferably rapid tests. | 2 | 2 | 0 | -4 | 3 |
| **20** | Decision scores and models perform better in making diagnostic decisions than GPs. | 1 | -4 | -1 | -1 | -2 |
| **21** | Healthcare is far behind in digitization compared to other sectors. | -2 | 0 | 0 | -1 | 4 |
| **22** | Digitization of care and diagnostics is moving faster than the current healthcare system can handle. | 0 | -2 | -1 | -1 | 1 |
| **23** | Healthcare providers should receive education aimed at dealing with Artificial Intelligence systems. | 0 | 1 | 2 | 0 | 4 |
| **24** | More attention should be paid to the professionalization of general practitioners with regard to dealing with technological developments. | -1 | -1 | 1 | -1 | 1 |
| **25** | The fragmentation of healthcare hinders the development of innovations in diagnostics. | -1 | 2 | -2 | 3 | 2 |
| **26** | The results of rapid tests performed in the general practice should be automatically entered into the hospital record. | 2 | 3 | 1 | 1 | 0 |
| **27** | Innovation of the diagnostic process should aim to move low complex second-line care to primary care. | 4 | -4 | 1 | -1 | -3 |
| **28** | The responsible use of Artificial Intelligence systems should be seen as a medical skill and should therefore be part of physician training. | 1 | 2 | 3 | 0 | 2 |
| **29** | A quality mark should be established for diagnostic innovations in family medicine. | -2 | -3 | 2 | -2 | 0 |
| **30** | A new funding system that is more focused on collaboration between primary and secondary care should be developed. | 4 | -3 | -2 | 4 | -1 |
| **31** | The government should be less reluctant to invest in diagnostic innovation. | -3 | 0 | 2 | 0 | -2 |
| **32** | Digital remote diagnostics should be more accessible to vulnerable groups. | 2 | -1 | -1 | 2 | -2 |
| **33** | A central digital healthcare platform gives patients more control over their own health. | 0 | 3 | -3 | -3 | -2 |
| **34** | Test results should be fed back to patients in understandable language. | 1 | 2 | 3 | 1 | 2 |
| **35** | The use of Artificial Intelligence in the consulting room is not good for the doctor-patient relationship. | -4 | -2 | -3 | 1 | -4 |
| **36*** | More rapid point-of-care tests should be available for use during a visit. | 0 | 1 | 0 | 0 | 1 |
| **37** | Deploying Artificial Intelligence-based systems speeds up and improves the diagnostic process. | 2 | -1 | 1 | 0 | 0 |
| **38** | Home measurements by patients are not reliable enough as diagnostics. | -4 | -3 | -1 | -4 | -3 |
| **39** | GPs should be better trained in the use of diagnostic tools. | -2 | -1 | 3 | 1 | 2 |
| **40** | The use of Artificial Intelligence systems is only suitable for simple diagnostic questions. | -3 | -1 | -2 | -1 | -1 |
| **41** | More effort should be put into the use of home measurements by patients. | 3 | 1 | -1 | 1 | 1 |
| **42** | High workload limits the implementation of innovation of diagnostics in primary care. | -2 | 0 | -2 | 0 | 3 |
| **43** | Digitization makes primary care more accessible and increases patient self-direction. | 1 | 1 | 1 | -2 | 1 |
| **44** | GPs need to collaborate more with data scientists and engineers to integrate Artificial Intelligence tools into primary care. | -1 | -1 | 2 | 1 | 0 |
| **45*** | Developers should collaborate more with healthcare providers and patients in the development of diagnostic innovations. | 1 | 2 | 0 | 2 | 1 |
| **46** | A different division of labor and way of working together in primary care is important for future-proof diagnostics in primary care. | -1 | 1 | -2 | 2 | 4 |
| **47** | Diagnostic innovation should ensure that patients can remain in primary care as much as possible. | 3 | -2 | 1 | -3 | -1 |
| **48** | Innovation should focus primarily on efficiency of the diagnostic process. | 0 | 0 | 3 | -2 | -1 |
| **49** | Artifical Intelligence should be used primarily to increase the efficiency of the diagnostic process. | -1 | 0 | 4 | -2 | 1 |
| **50** | There should be more focus on diagnostic innovation in general practitioner training. | 0 | 1 | 2 | 0 | 1 |
| **51** | An important factor in the success of a new diagnostic test is whether the health insurance company covers the cost of the test. | 0 | 2 | 3 | 1 | 0 |
| **52** | Diagnostic innovation should focus on cost savings. | -1 | -2 | 0 | -1 | -3 |
| **53** | The current funding system hinders innovation in the diagnostic process. | 1 | -1 | 0 | 3 | 0 |
| **54** | Digital remote diagnostics makes healthcare more accessible to patients. | 4 | 1 | -2 | -3 | -1 |
| **55** | Remote digital diagnostics causes inequities in access to care. | -4 | -2 | -3 | 2 | 0 |
| **56** | The interests of the patient must be at the center of the development and implementation of diagnostic innovations. | 0 | 4 | 2 | 1 | -1 |
| **57** | Technological developments such as Artificial Intelligence limit a physician's professional autonomy in making a diagnosis. | -3 | -3 | -4 | -4 | -3 |

**Consensus statements = statements that all perspectives agree or disagree with. These statements do not contribute to the distinction between the perspectives.*
